# Male Sex Is an Independent Predictor of Recurrence-Free Survival in Middle Eastern Papillary Thyroid Carcinoma

**DOI:** 10.3389/fendo.2022.777345

**Published:** 2022-03-10

**Authors:** Abdul K. Siraj, Sandeep Kumar Parvathareddy, Padmanaban Annaiyappanaidu, Nabil Siraj, Saif S. Al-Sobhi, Fouad Al-Dayel, Khawla S. Al-Kuraya

**Affiliations:** ^1^ Human Cancer Genomic Research, Research Center, King Faisal Specialist Hospital and Research Center, Riyadh, Saudi Arabia; ^2^ Department of Surgery, King Faisal Specialist Hospital and Research Center, Riyadh, Saudi Arabia; ^3^ Department of Pathology, King Faisal Specialist Hospital and Research Centre, Riyadh, Saudi Arabia

**Keywords:** papillary thyroid carcinoma, male sex, recurrence-free survival, prognosis, clinico-pathological associations

## Abstract

**Background:**

Disparity between sexes with regard to incidence, disease aggressiveness, and prognosis has been documented in several cancers. Although various reports have documented the association between male sex and aggressive papillary thyroid carcinoma (PTC), the prognostic impact of sex on PTC has been inconsistent. The role of sex in PTC aggressiveness and outcome in Middle Eastern PTC remains unknown. Therefore, our study retrospectively analyzed the data of a large cohort of Middle Eastern PTC patients to address this issue.

**Methods:**

We compared men and women with respect to clinico-pathological characteristics, disease persistence, structural recurrence, risk stratification, and prognosis. We included 1,430 patients—1,085 (75.9%) women and 345 (24.1%) men.

**Results:**

The median follow-up was 9.3 years. At diagnosis, 27% (93/345) of men were ≥55 years, compared with 17.8% (193/1085) of women (*p* = 0.0003). Men had significantly more advanced disease at presentation: higher stage (*p* = 0.0074), larger tumor size (*p* = 0.0069), higher rates of lymphovascular invasion (*p* = 0.0129), extrathyroidal extension (*p* = 0.0086), regional lymph node metastasis (*p* = 0.0279), and distant metastasis (*p* = 0.0101). There was a higher rate of recurrence (*p* < 0.0001) and *TERT* mutations (*p* = 0.0003) in male PTC patients than in female patients. Additionally, radioiodine refractoriness was higher in male PTC patients (*p* = 0.0014). In multivariate analysis, male sex was an independent prognostic factor for poor recurrence-free survival (RFS) (hazard ratio = 1.58; 95% confidence interval = 1.20–2.06; *p* = 0.0011).

**Conclusions:**

Men with PTC are more likely to present with more advanced and aggressive disease. Importantly, male sex was an independent prognostic factor for RFS. Thus, men may benefit from more aggressive management and therapeutic interventions.

## Introduction

Papillary thyroid carcinoma (PTC) is the most common endocrine malignancy (1). The incidence of PTC is on the rise over the past two decades (2, 3). In Saudi Arabia, PTC is the second commonest cancer affecting women, after breast cancer (4). PTC is an indolent disease with favorable prognosis in majority of patients. However, a subset of PTC patients (approximately one-third of all cases) will relapse (5–7), which could impact the quality of life for these patients. Thus, identification of clinical markers that could help to predict patients at high risk for recurrence is of great clinical importance for effective therapeutic interventions.

PTC is known to affect women more than men (1, 8). In fact, in Saudi Arabia, the age standardized incidence rate of thyroid cancer in women and men was 8.4 and 2.5 per 100,000 persons, respectively (4). The prognostic significance of sex in PTC remains controversial. While many studies have demonstrated the correlation between male sex and advanced stage, higher death rate, poor prognosis, and higher risk of recurrence in PTC (9–12), others have failed to identify prognostic difference between male and female PTCs when adjusting for age, stage, tumor size, and other influencing factors (13–15). Furthermore, current guidelines for risk stratification issued by the American Thyroid Association (ATA) does not include patient’s sex factor that could affect the risk of recurrence (16).

Despite the high incidence of PTC in Saudi Arabia and relatively high recurrence rate (17–19), the impact of sex on recurrence risk in PTC from Middle Eastern ethnicity remains unknown. Thus, we carried out this study on a large cohort of Middle Eastern adult PTC to investigate the impact of sex on clinico-pathological characteristics and patients’ prognosis. We also assess if male sex is an independent risk factor for recurrence of PTC from Middle Eastern ethnicity.

## Materials and Methods

### Patient Selection

One thousand four-hundred and thirty consecutive unselected adult PTC patients (≥18 years) diagnosed between 1988 and 2018 at King Faisal Specialist Hospital and Research Centre (Riyadh, Saudi Arabia) were included in the study. Cases were identified based on clinical history followed by fine needle aspiration cytology for confirmation. The Institutional Review Board of the hospital approved this study and the Research Advisory Council (RAC) provided waiver of consent under project RAC # 2110 031 and #2211168.

### Clinico-Pathological Data

Baseline clinico-pathological data were collected from case records and have been summarized in **Table 1**. Staging of PTC was performed using the eighth edition of the American Joint Committee on Cancer (AJCC) staging system. Based on the ATA guidelines, tall cell, hobnail, columnar cell, diffuse sclerosing, and insular variants were classified as aggressive variants, whereas classical and follicular variants were classified as non-aggressive variants (16). Prophylactic central lymph node dissection (PCLND) was performed in patients with clinically uninvolved central neck lymph nodes (cN0) who had either advanced primary tumors (T3 or T4) or clinically involved lateral neck nodes (cN1b), in accordance with the 2015 ATA guidelines (16). Of the 942 patients with cN0 PTC, 213 patients underwent PCLND. However, 343 patients who were eligible for PCLND did not undergo the procedure, based on the treating surgeon’s discretion. Only structural recurrence (local, regional, or distant) was considered for analysis. Recurrence was defined as any newly detected tumor (local or distant) or metastatic regional lymph node (LN), based on ultrasound and/or imaging studies in patients who had been previously free of disease following initial treatment. Regional lymph node metastases were further confirmed by cytological and/or histological examination. Persistent disease was defined as the presence of serum Tg at detectable levels, persisting/increasing Tg antibody levels, or occurrence of structural disease within 1 year after surgery. Localized PTC was defined as tumor confined to the thyroid without any extrathyroidal extension, LN metastasis, or distant metastasis, at the time of diagnosis. Radioactive iodine (RAI) refractory disease and risk categories were defined based on 2015 ATA guidelines (16).

**Table 1:** Patient characteristics for adult PTC (*n* = 1430).

	Total	
	No.	%
**Total**	1,430	
**Age at surgery (years)**		
Median (range)	39.2 (18.0–88.0)	
<55	1,144	80.0
≥55	286	20.0
**Gender**		
Female	1,085	75.9
Male	345	24.1
**Histologic subtype**		
Classical variant	943	66.0
Follicular variant	260	18.2
Tall cell variant	132	9.2
Other variants	95	6.6
**Tumor laterality**		
Unilateral	984	68.8
Bilateral	446	31.2
**Extrathyroidal extension**		
Present	609	42.6
Absent	821	57.4
**Multifocality**		
Yes	697	48.7
No	733	51.3
**Lymphovascular invasion**		
Present	296	20.7
Absent	1,134	79.3
**pT**		
T1	575	40.2
T2	463	32.4
T3	280	19.6
T4	112	7.8
**pN**		
N0	642	44.9
N1	685	47.9
Nx	103	7.2
**pM**		
M0	1,361	95.2
M1	69	4.8
**TNM Stage**		
I	1,171	81.9
II	163	11.4
III	22	1.5
IV	51	3.6
Unknown	23	1.6
***BRAF* mutation**		
Present	800	55.9
Absent	601	42.1
Unknown	29	2.0
***TERT* mutation**		
Present	197	13.8
Absent	1,120	78.3
Unknown	113	7.9
**Recurrence**		
Yes	260	18.2
No	1,170	81.8
**Disease persistence (after initial treatment)**		
No	1,023	71.5
Yes	407	28.5
**Disease persistence (at the end of follow-up)**		
No	1,366	95.5
Yes	64	4.5
**RAI given**		
Yes	1,188	80.7
No	242	19.3
**RAI refractory status**		
Refractory	235	19.8
Non-refractory	953	80.2
**ATA risk category**		
Low	234	16.4
Intermediate	500	35.0
High	696	48.7

### *BRAF* and *TERT* Mutation Analysis

*BRAF* and *TERT* mutation data were assessed in our laboratory by Sanger sequencing and have been published by us previously (20, 21).

### Follow-Up and Study Endpoint

Patients were regularly followed up by both physical examinations and imaging studies to identify tumor recurrence. The median follow-up was 9.3 years (range, 1.0–30.1 years). Recurrence-free survival (RFS) was defined as the time (in months) from date of initial surgery to the occurrence of any tumor recurrence (local, regional, or distant). In case of no recurrence, date of last follow-up was the study endpoint for RFS.

### Statistical Analysis

The associations between clinico-pathological variables and sex were performed using contingency table analysis and Chi square tests. Mantel–Cox log-rank test was used to evaluate RFS. Survival curves were generated using the Kaplan–Meier method. Cox proportional hazards model was used for multivariate analysis. Two-sided tests were used for statistical analyses with a limit of significance defined as *p*-value < 0.05. Data analyses were performed using the JMP14.0 (SAS Institute, Inc., Cary, NC) software package.

## Results

### Patient and Tumor Characteristics

Median age of the study population was 39.2 years (range: 18–88 years), with a male-to-female ratio of 1:3. The majority of tumors were classical variants of PTC (66.0%; 943/1,430); 31.2% (446/1,430) of tumors were bilateral and 48.7% (697/1,430) were multifocal; 42.6% (609/1,430) of tumors exhibited extrathyroidal extension and 20.7% (296/1,430) showed lymphovascular invasion. Tumor recurrence was seen in 18.2% (260/1,430) (**Table 1**).

### Clinico-Pathological Associations of Male Sex in PTC

In our cohort, 24.1% (345/1,430) of patients were male and 75.9% (1085/1430) were female. Male sex was associated with aggressive clinico-pathological characteristics such as older age (*p* = 0.0003), extrathyroidal extension (*p* = 0.0086), lymphovascular invasion (*p* = 0.0129), advanced T stage (*p* = 0.0069), LN metastasis (*p* = 0.0279), distant metastasis (*p* = 0.0101), and stage IV tumors (*p* = 0.0074). Male sex was also associated with tumor recurrence (*p* < 0.0001). To further corroborate the association of male sex with tumor recurrence, we analyzed the association of sex with ATA risk categories. Indeed, male sex was significantly associated with ATA high risk tumors (*p* = 0.0001). In addition, we found male sex to be associated with RAI refractoriness (*p* = 0.0014). Since *BRAF* and *TERT* mutation have been shown to be associated with RAI refractoriness, we sought to see if these mutations had a predilection for male sex. Although *TERT* mutation was associated with male sex (*p* = 0.0003), *BRAF* was not (*p* = 0.0938) (**Table 2**).

**Table 2:** Clinico-pathological associations of gender in papillary thyroid carcinoma.

	Male		Female		*p*-value
	No.	%	No.	%	
**Total**	345	24.1	1085	75.9	
**Age at surgery (years)**					
<55	252	73.0	892	82.2	0.0003
≥55	93	27.0	193	17.8	
**Histologic subtype**					
Aggressive variants	50	14.5	177	16.3	0.4163
Non-aggressive variants	295	85.5	908	83.7	
**Tumor laterality**					
Unilateral	223	64.6	761	70.1	0.0566
Bilateral	122	35.4	324	29.9	
**Extrathyroidal extension**					
Present	168	48.7	441	40.7	0.0086
Absent	177	51.3	644	59.3	
**Multifocality**					
Yes	162	47.0	535	49.3	0.4463
No	183	53.0	550	50.7	
**Lymphovascular invasion**					
Present	88	25.5	208	19.2	0.0129
Absent	257	74.5	877	80.8	
**pT**					
T1	126	36.5	449	41.4	0.0069
T2	99	28.7	364	33.5	
T3	87	25.2	193	17.8	
T4	33	9.6	79	7.3	
**pN**					
N0	134	43.0	508	50.0	0.0279
N1	178	57.0	507	50.0	
**pM**					
M0	319	92.5	1042	96.0	0.0101
M1	26	7.5	43	4.0	
**TNM Stage**					
I	259	77.3	912	85.1	0.0074
II	48	14.3	115	10.7	
III	6	1.8	16	1.5	
IV	22	6.6	29	2.7	
**RAI refractory status**					
Refractory	78	26.4	157	17.6	0.0014
Non-refractory	218	73.6	735	82.4	
**ATA risk category**					
Low	39	11.3	195	18.0	0.0001
Intermediate	106	30.7	394	36.3	
High	200	58.0	496	45.7	
***BRAF* mutation**					
Present	208	61.0	592	55.8	0.0938
Absent	133	39.0	468	44.2	
***TERT* mutation**					
Present	69	21.4	128	12.9	0.0003
Absent	253	78.6	867	87.1	
**Recurrence**					
Yes	92	26.7	168	15.5	<0.0001
No	253	73.3	917	84.5	

Since our cohort had a high rate of high-risk patients and we found a significant association between male sex and high-risk PTC, we sought to further analyze the clinico-pathological associations of male sex stratified by ATA risk categories. We found that male sex was associated with tumor recurrence in high-risk PTC (*p* = 0.0031), which stands true even on multivariate analysis (HR = 2.79, 95% CI = 1.45–5.76, *p* = 0.0016). In addition, male sex was associated with RAI refractoriness (*p* = 0.0085) and TERT mutation (*p* = 0.0341) in high-risk PTC. In intermediate-risk cases, male sex was found to be associated with other aggressive clinico-pathological characteristics, such as bilateral tumors (*p* = 0.0374) and advanced T stage (*p* = 0.0443). However, in both intermediate- and low-risk PTC, male sex was not associated with recurrence (**Supplementary Tables 1**–**3**).

### Recurrence Rate in Male and Female PTC Stratified by Stage

**Figure 1:**
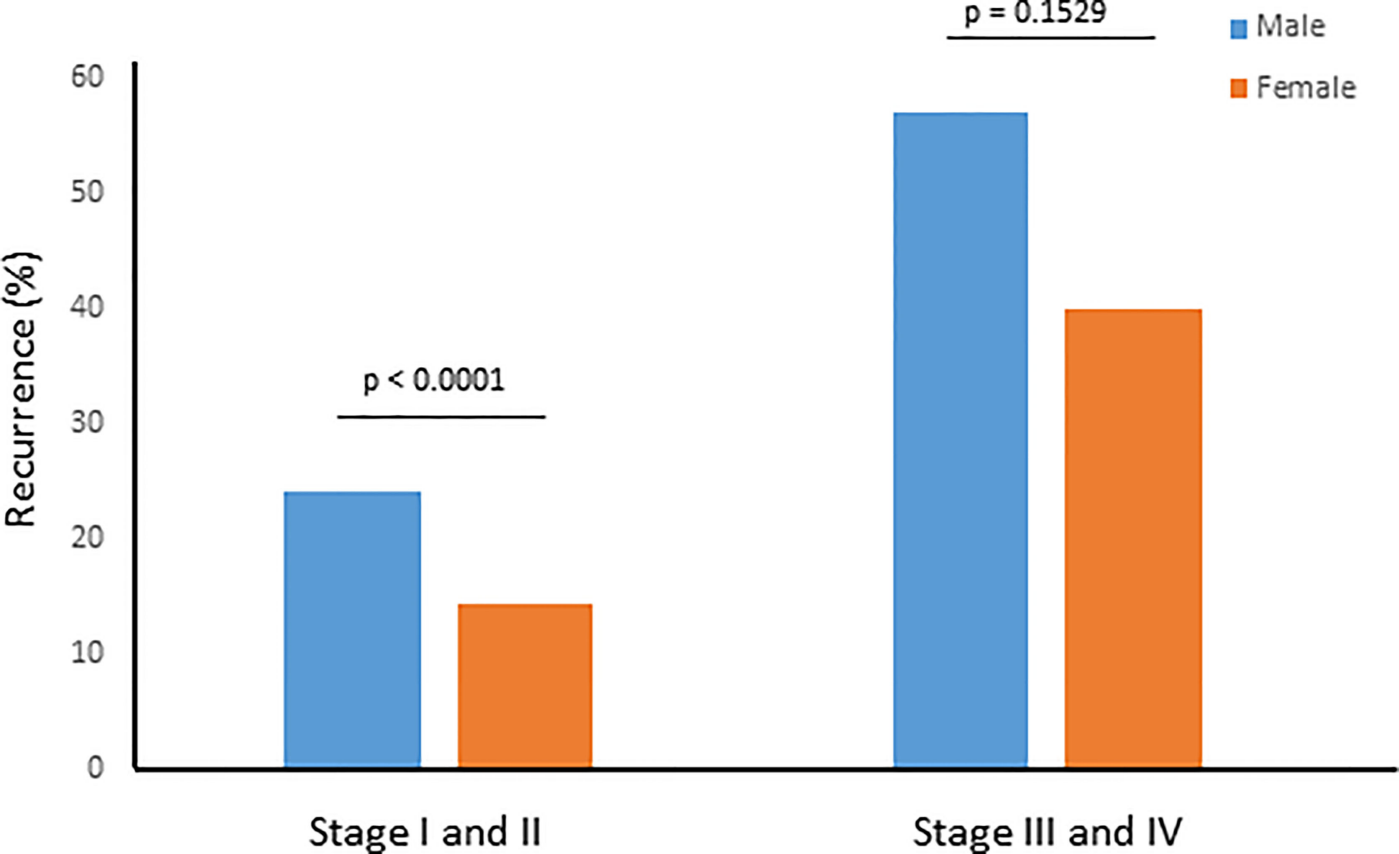
Recurrence rate for male and female sex stratified by tumor stage. Recurrence rate was significantly higher among men than women in early stage (I and II) tumors (24.2% vs. 14.4%; *p* < 0.0001), whereas the difference was not significant between male and female sex in late stage (III and IV) PTC (57.1% vs. 40.0%; *p* = 0.1529).

Since stage of tumor is an important prognostic factor, we sought to determine the recurrence rate in male and female PTCs among early-stage (stage I and II) and late-stage (stage III and IV) tumors. We found a significantly higher recurrence rate among men than women in early-stage tumors (24.2% vs. 14.4%; *p* < 0.0001), whereas the difference was not significant between male and female sex in late-stage PTC (57.1% vs. 40.0%; *p* = 0.1529) (**Figure 1**).

### Prognostic Significance of Sex in PTC

**Figure 2:**
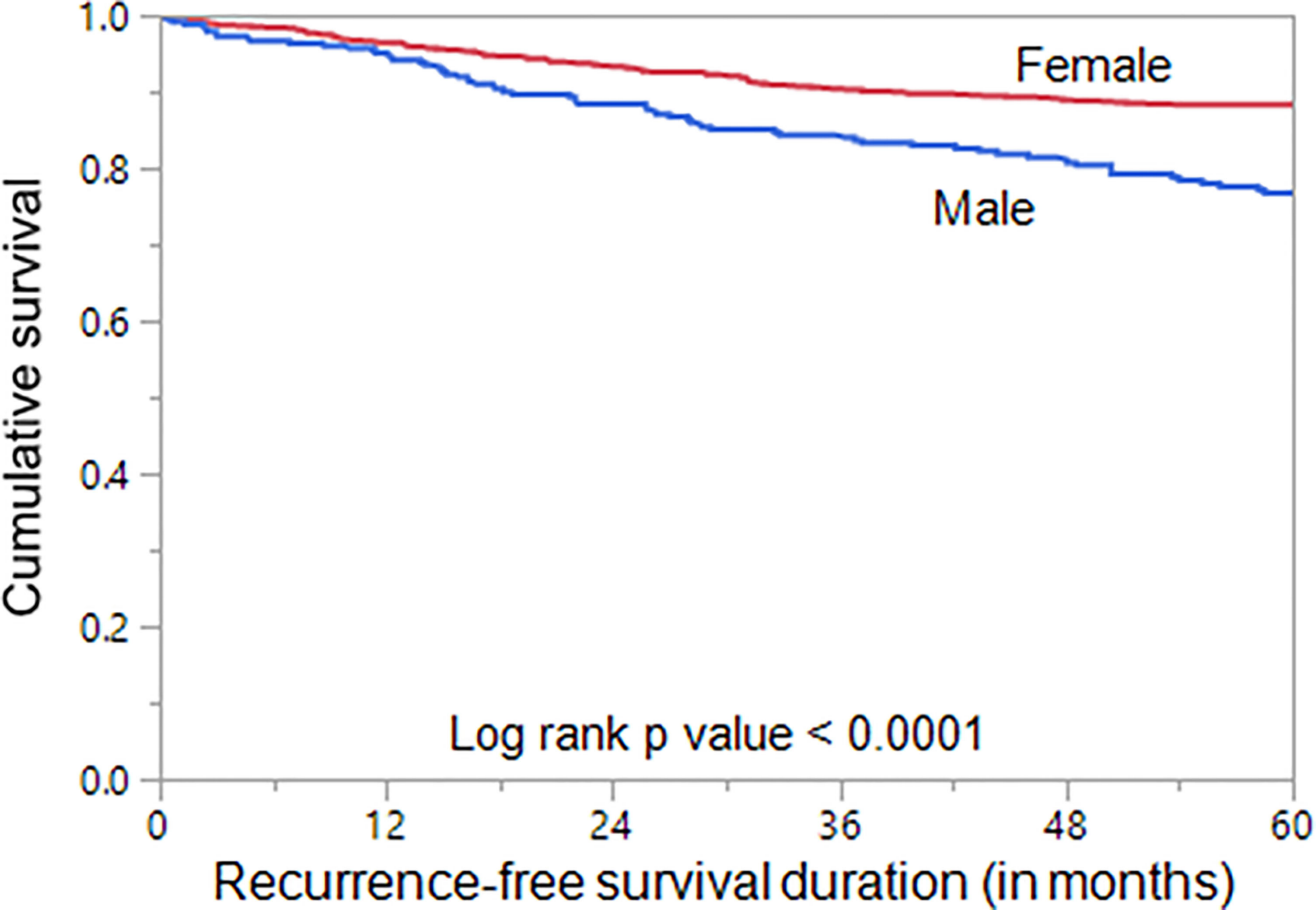
Sex and Recurrence-free survival. Kaplan–Meier survival curve showing poor recurrence-free survival in male sex compared to female sex (*p* < 0.0001).

We next analyzed the prognostic significance of sex in PTC. Male sex was associated with poor RFS (*p* < 0.0001; **Figure 2**). On multivariate analysis using Cox proportional hazards model, male sex was found to be an independent predictor of poor RFS (hazard ratio = 1.58; 95% confidence interval = 1.20–2.06; *p* = 0.0011), when adjusted for other clinico-pathological parameters. In addition, we also found age, tumor laterality, LN metastasis, distant metastasis, tumor stage, and ATA risk category to be independent predictors of RFS (**Table 3**).

**Table 3:** Multivariate analysis using Cox proportional hazard model for recurrence-free survival.

Clinico-pathological variables	Recurrence-free survival		
	Hazard ratio	95% Confidence interval	*p*-value
**Age**			
≥55 years (vs. <55 years)	2.66	1.91–3.64	<0.0001
**Sex**			
Male (vs. Female)	1.58	1.20–2.06	0.0011
**Histology**			
Aggressive variants (vs. non-aggressive variants)	0.98	0.66–1.41	0.9003
**Tumor laterality**			
Bilateral (vs. Unilateral)	1.56	1.03–2.44	0.0366
**Tumor focality**			
Multifocal (vs. Unifocal)	0.73	0.47–1.10	0.1325
**Extrathyroidal extension**			
Present (vs. Absent)	1.20	0.87–1.66	0.2616
**Lymphovascular invasion**			
Present (vs. Absent)	0.96	0.68–1.33	0.7948
**pT**			
T3–4 (vs. T1–2)	1.02	0.76–1.35	0.9093
**Lymph node metastasis**			
Present (vs. Absent)	1.76	1.31–2.40	0.0002
**Distant metastasis**			
Present (vs. Absent)	5.34	3.46–8.09	< 0.0001
**TNM stage**			
III–IV (vs. I–II)	0.50	0.28–0.86	0.0123
**ATA risk category**			
Low risk	Reference		
Intermediate risk	1.47	0.76–3.01	0.2579
High risk	2.79	1.45–5.76	0.0016

**Figure 3:**
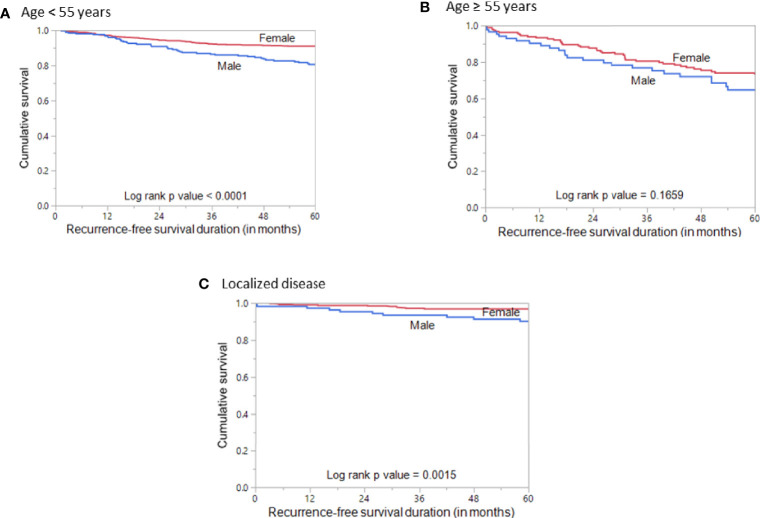
Recurrence-free survival stratified by age and disease localization. **(A)** Kaplan–Meier survival curve showing poor recurrence-free survival in male sex compared to female sex in patients aged < 55 years (*p* < 0.0001). **(B)** Kaplan–Meier survival curve showing no significant difference in recurrence-free survival between male and female sex in patients aged ≥ 55 years (*p* = 0.1659). **(C)** Kaplan–Meier survival curve showing poor recurrence-free survival in male sex compared to female sex in patients with localized PTC (*p* = 0.0015).

Since age is an important determinant of prognosis, we stratified the patients into younger age (<55 years) and older age (≥55 years) to analyze the prognostic differences with regard to sex. Interestingly, we found male sex to be associated with poor RFS (*p* < 0.0001; **Figure 3A**) only in the younger age PTCs but not in the older age PTCs (*p* = 0.1659; **Figure 3B**). We also analyzed the prognostic significance of sex in localized PTCs and found that male sex was associated with poor RFS (*p* = 0.0015; **Figure 3C**).

## Discussion

While sex disparity in the incidence of PTC and its clinical impact have been well documented, detailed analysis of prognostic impact of sex on PTC from Middle Eastern ethnicity has not been fully illustrated. Our study of more than 1,400 adult PTCs documented their clinico-pathological characteristics and demonstrated significantly more aggressive disease in men than women. The presence of extrathyroidal extension and lymphovascular invasion was seen significantly more commonly in men than women. There was a higher rate of distant metastasis and regional LN metastasis in men than women. Moreover, a higher rate of advanced disease (stage II, III, and IV according to the latest AJCC staging system) and a higher rate of patients with intermediate- or high-risk disease were also observed in male PTC patients. Interestingly, our subgroup analysis showed that aggressive PTC was more common in men even at young age. This further supports the notion that men inherently have more aggressive PTC behavior, which may not be attributable solely to delay in diagnosis. Several previous studies have shown the association between male sex and advanced disease (9, 10, 13, 22, 23).

Median age of the study population was 39.2 years. This finding is similar to studies from other Middle Eastern ethnicities (24–27), but lower than that seen in Western population (28). This most likely represents the inherent aggressive nature of PTC in the Middle Eastern ethnicity. Another consideration that needs to be taken into account is the age of the general population. In Saudi Arabia, the population pyramid is skewed toward younger age groups, showing a cone-shaped pattern. Indeed, nearly 60% of the Saudi population are under the age of 30 years. It has been shown previously that age of the population could partly explain the variability of age of onset for cancer, whereby younger populations tended to have a higher incidence of early-onset cancer (29, 30).

The rate of recurrence was 18.2% (260/1,430) in the overall cohort, which is relatively higher than what has been reported previously (31–33). The long follow-up duration and the low rate of PCLND may have contributed to the relatively high rate of recurrence in our study. The long median follow-up of 9.5 years might increase the likelihood to detect more recurrence in PTC patients than other studies with shorter median follow-up. In addition, PCLND has not been routinely performed in our center but rather it was performed based on tumor size and LN status, according to ATA guidelines (16). However, we have found that 343 PTC patients met the criteria for PCLND and yet did not undergo the procedure. In fact, 21.9% (75/343) of these patients developed recurrence during the follow-up period, which further contributed to the relatively high recurrence rate in our study. Furthermore, our study showed a higher recurrence risk in men than women. The recurrence risk was 1.7-fold higher in men presenting with AJCC stage I and II, compared to women. However, recurrence risk does not show significant difference between men and women for AJCC stage III and IV.

Our study findings showed that men had significantly poor RFS even in multivariate analysis where tumor stage and other influencing factors were considered. Moreover, significant prognostic differences between men and women was noted even when PTC was localized to the thyroid gland, which may suggest a truly aggressive PTC behavior in Middle Eastern men, even with small tumor size. Some previous studies have shown that sex was associated with poor prognosis (12, 23, 34, 35), whereas others found no prognostic difference between men and women (13–15). The lack of consensus among previous studies could be attributed to cohort size, classification used, and the inability to differentiate persistent disease from recurrent disease.

Another important finding is the significant association between male PTC and RAI refractoriness (RAIR). We attempted to explore the molecular features that might contribute to RAIR in male PTC patients, especially *BRAF* and *TERT* mutations. A large body of evidence have documented the correlation between *BRAF* and/or *TERT* mutation and poor RAI response (36–38). *BRAF* mutation data were available for 1,369 patients while *TERT* mutations were available in 1,288 patients of the study cohort. Interestingly, no correlation between *BRAF* mutations and male PTC patients was noted. However, a significantly higher rate of *TERT* mutations was observed in male PTC patients compared to female patients. A large meta-analyses, involving 32 studies, also found a significant association between *TERT* promoter mutations and male sex (39).

Our study has certain limitations. First, it has a historical, retrospective nature, it lacks complete information on some tumor features including *TERT* and *BRAF* mutations, and 3% of the patients were lost to follow-up. Second, the study cohort was limited to Middle Eastern ethnicity and it is from a single center that could slightly impact the generalizability of our results to other populations. Thirdly, our study population included a high rate of high-risk patients, which could be attributed to genetics or differences in presentation owing to the unique ethnicity. Whether this could also be partly attributed to delay in seeking healthcare remains to be explored.

In summary, our study demonstrated that men present with more advanced stage of disease, at older age and with higher rate of aggressive molecular and histopathological features. Men have a higher rate of recurrence and a shorter recurrence-free survival. Moreover, male gender was an independent prognostic factor for RFS in Middle Eastern PTC patients. Overall, the results of this study strongly suggest that sex should be considered as an important predictor of prognosis and therefore men with PTC may benefit from more aggressive initial treatment and intense follow-up.

## Data Availability Statement

The original contributions presented in the study are included in the article/**Supplementary Material**. Further inquiries can be directed to the corresponding author.

## Ethics Statement

The studies involving human participants were reviewed and approved by the Research Ethics Committee, King Faisal Specialist Hospital and Research Centre. Written informed consent for participation was not required for this study in accordance with the national legislation and the institutional requirements.

## Author Contributions

Study concept and design: KA-K, SP, and AS. Executed the study: SP, AS, PA, NS, SA-S, and FA-D. Statistical analysis: SP. Drafting the article: KA-K, AS, and SP. Critical revision of the article for important intellectual content, writing of the article, and approval of the final version: KA-K, SP, AS, PA, NS, SA-S, and FA-D. All authors contributed to the article and approved the submitted version.

## Conflict of Interest

The authors declare that the research was conducted in the absence of any commercial or financial relationships that could be construed as a potential conflict of interest.

## Publisher’s Note

All claims expressed in this article are solely those of the authors and do not necessarily represent those of their affiliated organizations, or those of the publisher, the editors and the reviewers. Any product that may be evaluated in this article, or claim that may be made by its manufacturer, is not guaranteed or endorsed by the publisher.
