# Supplementary material for: Male Sex Is an Independent Predictor of Recurrence-Free Survival in Middle Eastern Papillary Thyroid Carcinoma
Source: Front Endocrinol (Lausanne). 2022 Mar 10;13:777345. doi: 10.3389/fendo.2022.777345 (PMC8959980; doi:10.3389/fendo.2022.777345)
Supplement: Supplementary file 1 [file DataSheet_1.docx]

**Supplementary Table 1.** Clinico-pathological associations of gender in low-risk papillary thyroid carcinoma

|  | **Male** | | **Female** | | **p value** |
| --- | --- | --- | --- | --- | --- |
|  | No. | % | No. | % |  |
| **Total** | 39 | 16.7 | 195 | 83.3 |  |
| **Age at surgery (years)** |  |  |  |  |  |
| < 55 | 29 | 74.4 | 173 | 88.7 | 0.0266 |
| ≥ 55 | 10 | 25.6 | 22 | 11.3 |  |
| **Tumor laterality** |  |  |  |  |  |
| Unilateral | 29 | 74.4 | 150 | 76.9 | 0.7324 |
| Bilateral | 10 | 25.6 | 45 | 23.1 |  |
| **Multifocality** |  |  |  |  |  |
| Yes | 16 | 41.0 | 85 | 43.6 | 0.7675 |
| No | 23 | 59.0 | 110 | 56.4 |  |
| **pT** |  |  |  |  |  |
| T1 | 35 | 89.7 | 180 | 92.3 | 0.2863 |
| T2 | 2 | 5.1 | 13 | 6.7 |  |
| T3 | 2 | 5.1 | 2 | 1.0 |  |
| **pN** |  |  |  |  |  |
| N0 | 29 | 82.9 | 151 | 80.3 | 0.7268 |
| N1 | 6 | 17.1 | 37 | 19.7 |  |
| **TNM Stage** |  |  |  |  |  |
| I | 32 | 82.1 | 156 | 80.4 | 0.8130 |
| II | 7 | 17.9 | 38 | 19.6 |  |
| **RAI refractory status** |  |  |  |  |  |
| Refractory | 2 | 6.9 | 3 | 2.3 | 0.2446 |
| Non-refractory | 27 | 93.1 | 128 | 97.7 |  |
| ***BRAF* mutation** |  |  |  |  |  |
| Present | 19 | 50.0 | 79 | 41.6 | 0.3403 |
| Absent | 19 | 50.0 | 111 | 58.4 |  |
| ***TERT* mutation** |  |  |  |  |  |
| Present | 1 | 3.0 | 11 | 6.4 | 0.4154 |
| Absent | 32 | 97.0 | 161 | 93.6 |  |
| **Recurrence** |  |  |  |  |  |
| Yes | 4 | 10.3 | 8 | 4.1 | 0.1467 |
| No | 35 | 89.7 | 187 | 95.9 |  |

**Supplementary Table 2.** Clinico-pathological associations of gender in intermediate-risk papillary thyroid carcinoma

|  | **Male** | | **Female** | | **p value** |
| --- | --- | --- | --- | --- | --- |
|  | No. | % | No. | % |  |
| **Total** | 106 | 21.2 | 394 | 78.8 |  |
| **Age at surgery (years)** |  |  |  |  |  |
| < 55 | 89 | 84.0 | 350 | 88.8 | 0.1864 |
| ≥ 55 | 17 | 16.0 | 44 | 11.2 |  |
| **Histologic subtype** |  |  |  |  |  |
| Aggressive variants | 14 | 13.2 | 65 | 16.5 | 0.4015 |
| Non-aggressive variants | 92 | 86.8 | 329 | 83.5 |  |
| **Tumor laterality** |  |  |  |  |  |
| Unilateral | 69 | 65.1 | 297 | 75.4 | 0.0374 |
| Bilateral | 37 | 34.9 | 97 | 24.6 |  |
| **Extrathyroidal extension** |  |  |  |  |  |
| Present | 25 | 23.6 | 65 | 16.5 | 0.1003 |
| Absent | 81 | 76.4 | 329 | 83.5 |  |
| **Multifocality** |  |  |  |  |  |
| Yes | 48 | 45.3 | 179 | 45.4 | 0.9783 |
| No | 58 | 54.7 | 215 | 54.6 |  |
| **Lymphovascular invasion** |  |  |  |  |  |
| Present | 27 | 25.5 | 88 | 22.3 | 0.4995 |
| Absent | 79 | 74.5 | 306 | 77.7 |  |
| **pT** |  |  |  |  |  |
| T1 | 42 | 39.6 | 132 | 33.5 | 0.0443 |
| T2 | 54 | 50.9 | 245 | 62.2 |  |
| T3 | 10 | 9.4 | 17 | 4.3 |  |
| **pN** |  |  |  |  |  |
| N0 | 36 | 40.0 | 145 | 42.4 | 0.6812 |
| N1 | 54 | 60.0 | 197 | 57.6 |  |
| **TNM Stage** |  |  |  |  |  |
| I | 94 | 89.5 | 364 | 93.3 | 0.2158 |
| II | 11 | 10.5 | 24 | 6.2 |  |
| III | 0 | 0.0 | 0 | 0.0 |  |
| IV | 0 | 0.0 | 2 | 0.5 |  |
| **RAI refractory status** |  |  |  |  |  |
| Refractory | 15 | 16.0 | 47 | 14.5 | 0.7210 |
| Non-refractory | 79 | 84.0 | 278 | 85.5 |  |
| ***BRAF* mutation** |  |  |  |  |  |
| Present | 57 | 54.3 | 199 | 51.4 | 0.6021 |
| Absent | 48 | 45.7 | 188 | 48.6 |  |
| ***TERT* mutation** |  |  |  |  |  |
| Present | 12 | 12.0 | 19 | 5.1 | 0.0215 |
| Absent | 88 | 88.0 | 352 | 94.9 |  |
| **Recurrence** |  |  |  |  |  |
| Yes | 14 | 13.2 | 33 | 8.4 | 0.1450 |
| No | 92 | 86.8 | 361 | 91.6 |  |

**Supplementary Table 3.** Clinico-pathological associations of gender in high-risk papillary thyroid carcinoma

|  | **Male** | | **Female** | | **p value** |
| --- | --- | --- | --- | --- | --- |
|  | No. | % | No. | % |  |
| **Total** | 200 | 28.7 | 496 | 71.3 |  |
| **Age at surgery (years)** |  |  |  |  |  |
| < 55 | 134 | 67.0 | 369 | 74.4 | 0.0509 |
| ≥ 55 | 66 | 33.0 | 127 | 25.6 |  |
| **Histologic subtype** |  |  |  |  |  |
| Aggressive variants | 36 | 18.0 | 112 | 22.6 | 0.1814 |
| Non-aggressive variants | 164 | 82.0 | 384 | 77.4 |  |
| **Tumor laterality** |  |  |  |  |  |
| Unilateral | 125 | 62.5 | 314 | 63.3 | 0.8420 |
| Bilateral | 75 | 37.5 | 182 | 36.7 |  |
| **Extrathyroidal extension** |  |  |  |  |  |
| Present | 142 | 71.0 | 365 | 73.6 | 0.4889 |
| Absent | 58 | 29.0 | 131 | 26.4 |  |
| **Multifocality** |  |  |  |  |  |
| Yes | 98 | 49.0 | 271 | 54.6 | 0.1778 |
| No | 102 | 51.0 | 225 | 45.4 |  |
| **Lymphovascular invasion** |  |  |  |  |  |
| Present | 60 | 30.0 | 109 | 22.0 | 0.0275 |
| Absent | 140 | 70.0 | 387 | 78.0 |  |
| **pT** |  |  |  |  |  |
| T1 | 49 | 24.5 | 137 | 27.6 | 0.8520 |
| T2 | 43 | 21.5 | 106 | 21.4 |  |
| T3 | 75 | 37.5 | 174 | 35.1 |  |
| T4 | 33 | 16.5 | 79 | 15.9 |  |
| **pN** |  |  |  |  |  |
| N0 | 63 | 33.7 | 175 | 36.1 | 0.5602 |
| N1 | 124 | 66.3 | 310 | 63.9 |  |
| **pM** |  |  |  |  |  |
| M0 | 174 | 87.0 | 453 | 91.3 | 0.0836 |
| M1 | 26 | 13.0 | 43 | 8.7 |  |
| **TNM Stage** |  |  |  |  |  |
| I | 126 | 66.0 | 354 | 72.5 | 0.0681 |
| II | 37 | 19.4 | 91 | 18.7 |  |
| III | 6 | 3.1 | 16 | 3.3 |  |
| IV | 22 | 11.5 | 29 | 5.5 |  |
| **RAI refractory status** |  |  |  |  |  |
| Refractory | 61 | 35.3 | 107 | 24.5 | 0.0085 |
| Non-refractory | 112 | 64.7 | 329 | 75.5 |  |
| ***BRAF* mutation** |  |  |  |  |  |
| Present | 132 | 66.7 | 314 | 65.0 | 0.6792 |
| Absent | 66 | 33.3 | 169 | 35.0 |  |
| ***TERT* mutation** |  |  |  |  |  |
| Present | 56 | 29.6 | 98 | 21.7 | 0.0341 |
| Absent | 133 | 70.4 | 354 | 78.3 |  |
| **Recurrence** |  |  |  |  |  |
| Yes | 74 | 37.0 | 127 | 25.6 | 0.0031 |
| No | 126 | 63.0 | 369 | 74.4 |  |
